# Dynamics of the Toxin Cylindrospermopsin and the Cyanobacterium *Chrysosporum* (*Aphanizomenon*) *ovalisporum* in a Mediterranean Eutrophic Reservoir

**DOI:** 10.3390/toxins6113041

**Published:** 2014-10-28

**Authors:** Ali Fadel, Ali Atoui, Bruno J. Lemaire, Brigitte Vinçon-Leite, Kamal Slim

**Affiliations:** 1 Laboratory of Microorganisms and Food Irradiation, Lebanese Atomic Energy Commission-CNRS, P.O. Box 11-8281, Riad El Solh, Beirut 1107 2260, Lebanon; E-Mail: a.atoui@cnrs.edu.lb; 2 LEESU (UMR MA-102) Université Paris-Est, Ecole des Ponts ParisTech, AgroParisTech, Marne-la-Vallée F-77455, France; E-Mails: ali.fadel@leesu.enpc.fr (A.F.); bruno.lemaire@leesu.enpc.fr (B.J.L.); bvl@leesu.enpc.fr (B.V.-L.); 3 Department of Forest, Water and Environmental Sciences and Engineering, AgroParisTech, Paris F-75005, France

**Keywords:** cyanobacteria, water temperature, Middle East, solar radiation, nutrients

## Abstract

*Chrysosporum ovalisporum* is a cylindrospermopsin toxin producing cyanobacterium that was *reported in several lakes and reservoirs. *Its growth dynamics and toxin distribution in field remain largely undocumented. *Chrysosporum ovalisporum* was reported in 2009 in Karaoun Reservoir, Lebanon. We investigated the factors controlling the occurrence of this cyanobacterium and vertical distribution of cylindrospermopsin in Karaoun Reservoir. We conducted bi-weekly sampling campaigns between May 2012 and August 2013. Results showed that *Chrysosporum ovalisporum* is an ecologically plastic species that was observed in all seasons. Unlike the high temperatures, above 26 °C, which is associated with blooms of *Chrysosporum ovalisporum* in Lakes Kinneret (Israel), Lisimachia and Trichonis (Greece) and Arcos Reservoir (Spain), *Chrysosporum ovalisporum* in Karaoun Reservoir bloomed in October 2012 at a water temperature of 22 °C during weak stratification. Cylindrospermopsin was detected in almost all water samples even when *Chrysosporum ovalisporum* was not detected. *Chrysosporum ovalisporum* biovolumes and cylindrospermopsin concentrations were not correlated (*n* = 31, *r*^2^ = −0.05). Cylindrospermopsin reached a maximum concentration of 1.7 µg L^−1^. The vertical profiles of toxin concentrations suggested its possible degradation or sedimentation resulting in its disappearance from the water column. The field growth conditions of *Chrysosporum ovalisporum* in this study revealed that it can bloom at the subsurface water temperature of 22 °C increasing the risk of its development and expansion in lakes located in temperate climate regions.

## 1. Introduction

Many lakes and reservoirs throughout the world suffer from toxic cyanobacterial blooms e.g., [1,2,3,4,5]. *Chrysosporum ovalisporum*, previously known as *Aphanizomenon ovalisporum* [6] is a toxic bloom-forming cyanobacterium that was reported in several freshwater bodies mainly in Australia and around the Mediterranean Sea [7,8,9,10]. Chrysosporum ovalisporum is a planktonic nostocalean that can colonize freshwater bodies due to different competitive strategies. Its eco-physiological characteristics are presented in Table 1. In a stratified water column, its gas vacuoles enable it to migrate between surface layers with high light availability and deeper layers with high nutrient availability [11]. Its colonies are characterized by thick wall cells called heterocysts, dedicated to atmospheric nitrogen fixation during nitrogen limitation periods [12]. Moreover, its filamentous morphology and colony size offer protection against grazing [13].

**Table 1:** Eco-physiological characteristics of *Chrysosporum ovalisporum*.

Parameter	*Chrysosporum ovalisporum*
Laboratory optimal growth temperature (°C)	28 ± 2 ^a^
	33 ± 2 ^b^
	32.8 ± 0.9 ^c^
	26 ± 1 ^d^
Maximum growth rate at optimal temperature (day^−1^)	0.3 ^a^
	0.36 ^c^
Filament flotation rate (m h^−1^)	<0.04 ^e^
Optimal solar irradiation (W m^−2^)	80 ^a^

Source: ^a^ [14]; ^b^ [15]; ^c^ [16]; ^d^ [9]; ^e^ [17].

*Chrysosporum ovalisporum* produces cylindrospermopsin (CYN), a toxin that poses serious threats to human and environmental health. CYN is produced by some cyanobacterial species other than *Chrysosporum ovalisporum* including Chrysosporum (*Anabaena*) *bergii*, *Cylindrospermopsis raciborskii*, *Raphidiopsis curvata*, and *Umezakia natans* [18,19]. This toxin, produced by *Cylindrospermopsis raciborskii* is believed to be responsible for the severe hepatoenteritis that affected 148 people in 1979 on Palm Island, Queensland, Australia [20]. CYN is a water soluble alkaloid hepatotoxin that was found to cause damage to kidneys, lungs and heart. It was also reported as protein synthesis inhibitor, genotoxic [21] and carcinogenic [22].

A large fraction of CYN can be found in extracellular water because it is released from cells under physiological stress by temperature and light [23]. CYN persists in many water bodies because of its chemical stability and slow degradation; after 10 weeks at 50 °C, cylindrospermopsin had degraded to 57% of the original concentration [24]. Recently, it was found in high extracellular concentrations in many freshwater bodies throughout the world: 18.4 µg L^−^^1^ in Lake Albano, Italy [25], 9.4 µg L^−^^1^ by *Chrysosporum ovalisporum* in Arcos Reservoir, Spain [9] and 12.1 µg L^−^^1^ in German lakes [26].

Understanding the mechanisms and processes that control the growth and succession of cyanobacterial species is of great concern. Karaoun Reservoir is the largest freshwater body in Lebanon, with a maximum capacity of 224 × 10^6^ m^3^. Before 1996, the reservoir was dominated by diatoms that constituted 80% of the total population [27]. After the year 2000, the dinoflagellate *Ceratium hirundinella* and filamentous green algae were the main phytoplankton species documented in the reservoir [28]. *Chrysosporum ovalisporum* blooms were first reported in Karaoun Reservoir in May 2009 [29,30]. *Chrysosporum ovalisporum* is not as widely spread as other cyanobacteria species like *Microcystis aeruginosa*. It was documented in some water bodies around the Mediterranean Sea but its growth dynamics were not sufficiently studied. As well, cylindrospermopsin toxin profiles were poorly studied at field. In this paper, we describe the dynamics and controlling factors of this blooming cyanobacterium as well as CYN distribution in the water column of Karaoun Reservoir.

## 2. Results

### 2.1. Physical-Chemical Conditions

**Figure 1:**
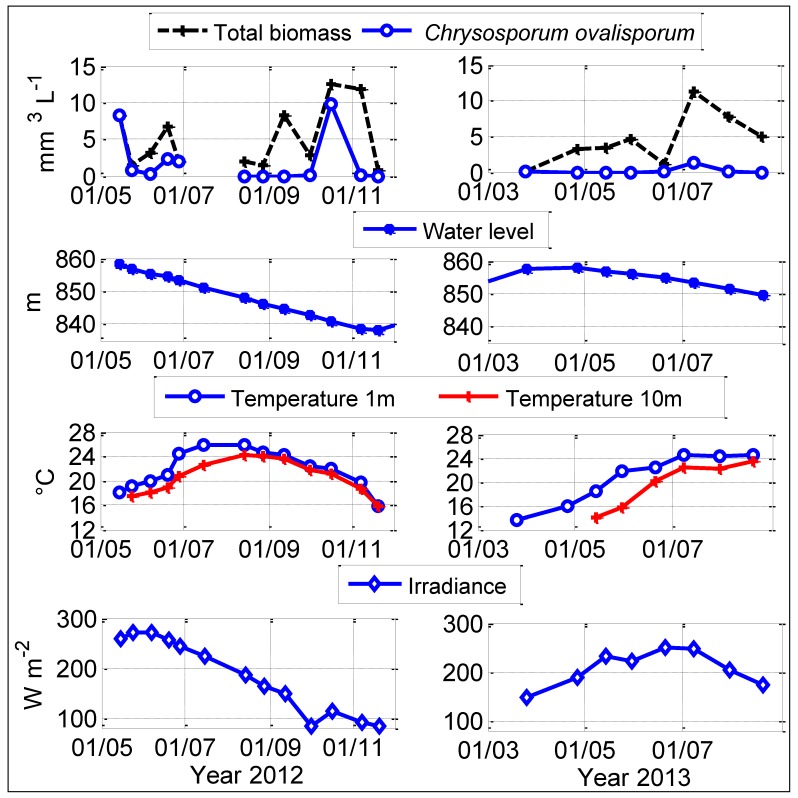
Daily mean values of physical variables at the sampling dates: water level, solar irradiance, water temperature at 1 and 10 m, and biovolumes of total phytoplankton and *Chrysosporum ovalisporum* in 2012 and 2013 at S_M_ in Karaoun Reservoir, except on May 15, 2012 where samples were taken at S_B_.

**Figure 2:**
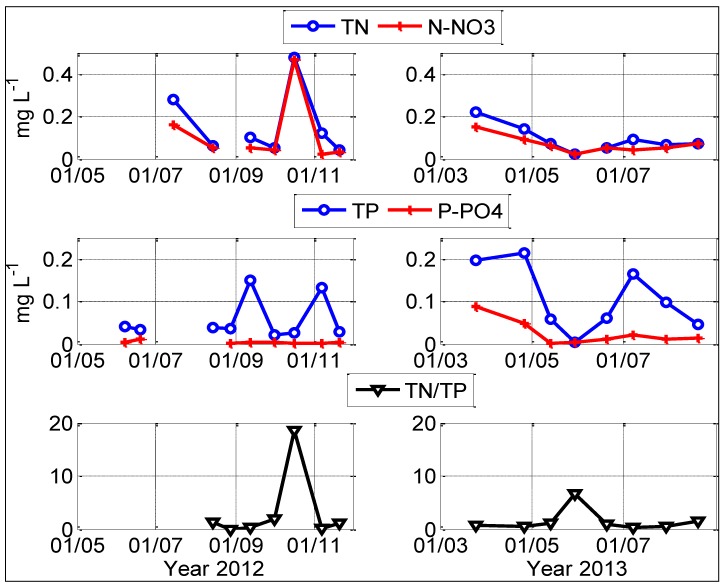
Concentrations of nitrate nitrogen, total nitrogen, total phosphorus, orthophosphate phosphorus and *TN*/*TP* ratio in 2012 and 2013 at S_M_ in Karaoun Reservoir.

During 2012 and 2013, subsurface water temperature in Karaoun Reservoir ranged from 13 to 26 °C (Figure 1). Comparison between water temperatures at 1 and 10 m in 2012 and 2013 showed that the reservoir was stratified from May to August. The water was weakly stratified (less than 1 °C between the surface and the lake bottom) or fully mixed in September, October and November 2012. The water level ranged from 837 to 859 m above sea level. The reservoir was full at the beginning of May in 2012 and 2013. Then, the water level decreased by 20 m due to small inflows and regular withdrawals in the dry season, between May and October. Subsurface orthophosphate concentration was close to detection limit (0.01 mg P L^−1^) in 2012. In 2013, it decreased from 0.95 mg L^−1^ in March down to under the detection limit in summer. Total phosphorus varied greatly between campaigns and some of its peaks were correlated with total phytoplankton biovolume peaks. Nitrate nitrogen did not exceed 0.2 mg L^−^^1^ except on October 16, 2012 (0.47 mg L^−1^) (Figure 2). TN:TP ratio did not exceed 22:1 during the study period.

### 2.2. Dynamics of Chrysosporum ovalisporum in Karaoun Reservoir

**Figure 3:**
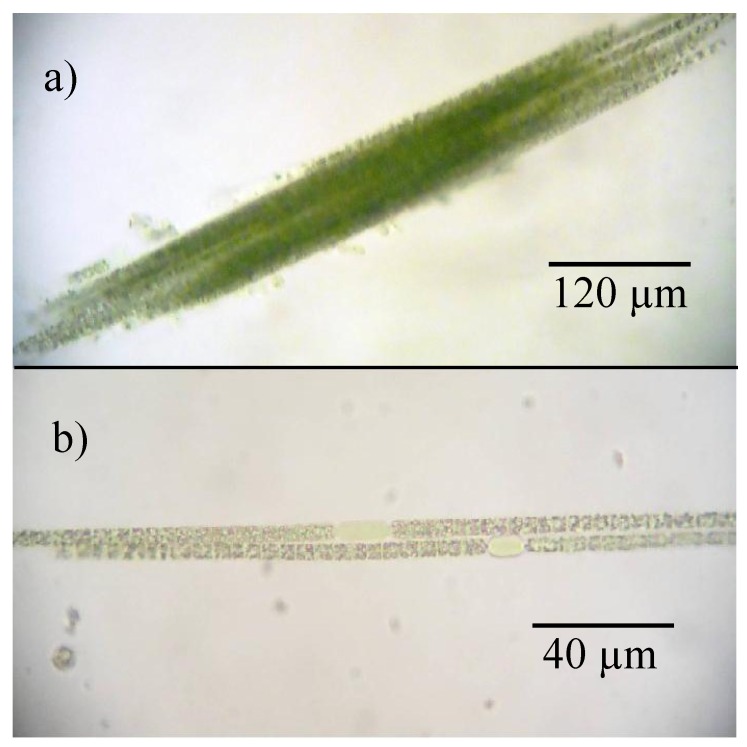
*Chrysosporum ovalisporum* at Karaoun Reservoir (**a**) colony on October 16, 2012; (**b**) visible heterocyst on March 25, 2013.

*Chrysosporum ovalisporum* in Karaoun Reservoir was already blooming at the beginning of the survey on May 15, 2012 with a biovolume of 8.2 mm^3^ L^−1^ in a sample taken at the edge of the reservoir (S_B_, see section 4.1.). At that time, the reservoir was full. This bloom had declined a week after the water level had begun to decrease on May 24, 2012. *Chrysosporum ovalisporum* bloomed again in June but disappeared in July. Subsurface nitrate nitrogen concentration was 0.16 mg N L^−1^ and water temperature was 25 °C at that time. *Chrysosporum ovalisporum* was not detected from August to September 2012 when the reservoir was dominated by *Microcystis aeruginosa* that had a maximum biovolume of 6.7 mm^3^ L^−1^ on September 12, 2012. In mid-October 2012, *Microcystis aeruginosa* was not detected and was replaced by *Chrysosporum ovalisporum* colonies with trichomes of 150 ± 75 μm without heterocysts (Figure 3a). It was a mixing period, orthophosphate concentration was close to detection limit (0.01 mg P L^−1^), nitrate concentration was 0.47 mg N L^−1^, water level was low (10 m depth at S_M_, 841 m above sea level), daily average irradiance was 120 W m^−2^ and water temperature was 22 °C. After 2 weeks, *Chrysosporum ovalisporum* was not detected anymore and was replaced by dinoflagellate *Ceratium hirundinella* which bloomed in November.

In 2013, *Chrysosporum ovalisporum* was observed in March and July but its biovolumes did not exceed 1.3 mm^3^ L^−1^. In March, *Chrysosporum ovalisporum* trichomes of 130 ± 50 μm showed visible heterocysts (Figure 3b), while nitrate nitrogen concentration was 0.15 mg N L^−1^.

In summary, during both years, *Chrysosporum ovalisporum* was seen both at high and low water levels, during stratified and unstratified conditions, in a wide range of daily average irradiance (100–260 W m^−2^).

**Figure 4:**
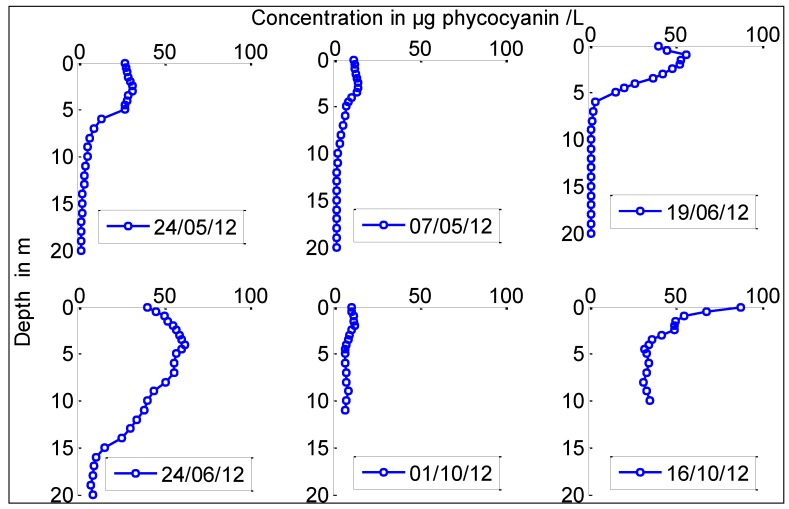
Phycocyanin fluorescence profiles, proxies of *Chrysosporum ovalisporum* concentration in the water column at S_M_ in Karaoun Reservoir in 2012 and 2013.

Figure 4 presents the phycocyanin profiles of *Chrysosporum ovalisporum* measured in 2012 and 2013. The relative proportion of each cyanobacterial species with respect to the total biovolume of cyanobacteria group was calculated using microscopic counting. This proportion was then used to calculate their corresponding phycocyanin values measured by Trios fluoremeter. Phycocyanin profiles showed the seasonal variation of *Chrysosporum ovalisporum* profiles. In late spring, May and June 2012, when daily irradiance ranged between 230 and 270 W m^−2^, *Chrysosporum ovalisporum* was present in the top 5 m, in the euphotic zone of Karaoun Reservoir, and was concentrated between 1 and 3 m. In autumn, in October 2012, when irradiance was 100 ± 20 W m^−2^, *Chrysosporum ovalisporum* exhibited a surface bloom.

### 2.3. Cylindrospermopsin Quantification

**Figure 5:**
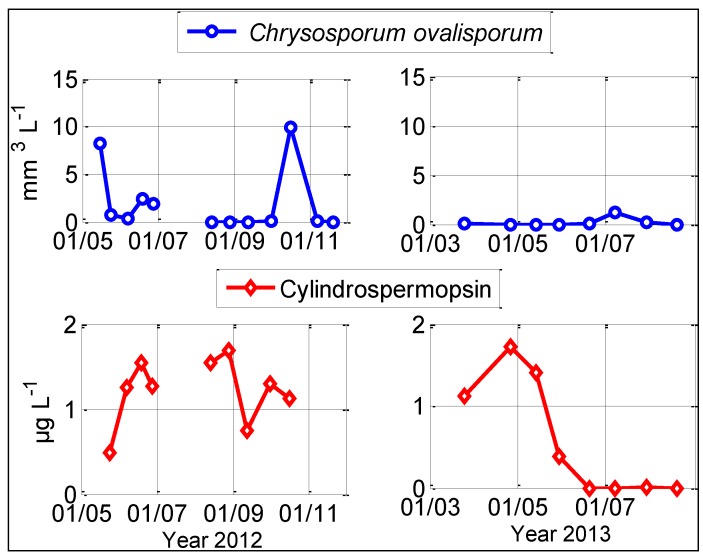
Subsurface cylindrospermopsin (CYN) concentration and biovolumes of *Chrysosporum ovalisporum* in 2012 and 2013 at S_M_ in Karaoun Reservoir, except on May 15, 2012 where sample were taken at S_B_.

Subsurface concentrations of CYN in Karaoun Reservoir ranged from 0.38 to 1.72 µg L^−1^ in 2012 and 2013 (Figure 5). The lowest concentration (0.38 µg L^−1^) was recorded at the beginning of a *Chrysosporum ovalisporum* bloom on May 15, 2012. This concentration showed an increasing trend in the first four campaigns (May 15, May 24, June 7 and June 19, 2012). The highest concentration (1.7 µg L^−1^) was recorded both on August 28, 2012 and April 26, 2013, in the absence of *Chrysosporum ovalisporum* in the water column.

### 2.4. Comparison Between Chrysosporum ovalisporum and CYN Distribution in the Water Column

**Figure 6:**
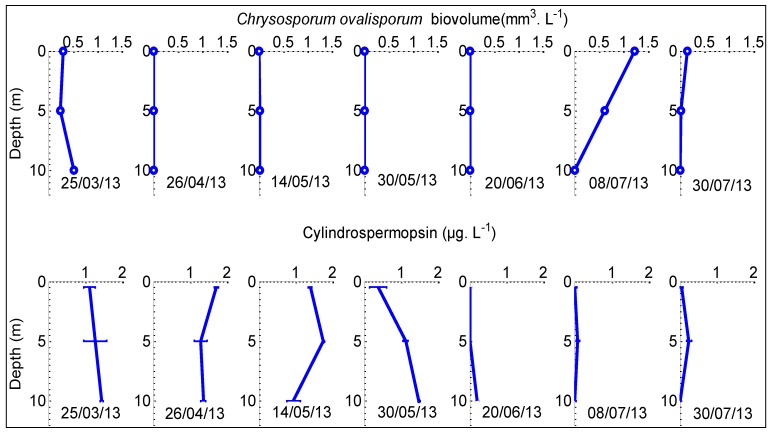
Vertical profiles of *Chrysosporum ovalisporum* biovolumes (10^−3^ mm^3^ L^−1^) and CYN concentrations (µg L^−1^) in Karaoun Reservoir during the year 2013. Error-bars are the standard deviations on the runned triplicates.

*Chrysosporum ovalisporum* was the only CYN producing cyanobacteria species recorded in the reservoir in both 2012 and 2013. We therefore tried to compare the distribution of the cyanobacterium and of the toxin in the water column. In 2013, *Chrysosporum ovalisporum* was first observed on March 25. It was not detected in April, May and June 2013 but it was detected again in July 2013 at a low biovolume (Figure 6). On March 25, 2013, CYN concentrations were higher than 1 µg L^−1^ at the surface and at 5 m and 10 m depths. In April 2013, the CYN concentration increased from 1.1 to 1.7 µg L^−1^ at the surface and remained constant at 5 m and 10 m depth. On May 14, 2013, it decreased from 1.7–1.4 µg L^−1^ at the surface and from 1.3 to 0.9 µg L^−1^ at 10 m and increased from 1.29 to 1.7 µg L^−1^ at 5 m. On May 30, 2013, it decreased from 1.4 to 0.38 µg L^−1^ at the surface and from 1.7 to 1.1 µg L^−1^ at 5 m depth. This decrease at 1 and 5 m was accompanied by an increase from 0.9 to 1.5 µg L^−1^ at 10 m depth. CYN was not detected down to 5 m depth on June 20, 2013 and was at a low concentration of 0.2 µg L^−1^ at 10 m depth. On July 8, 2013, CYN was not detected at the surface and 10 m and was 0.09 µg L^−1^ at 5 m depth. On July 30, 2013 following *Chrysosporum ovalisporum* detection on July 8, 2013, CYN increased only at 5 m, from 0.09 to 0.25 µg L^−1^.

### 2.5. Absence of Correlation between Cylindrospermopsin Concentration and Chrysosporum ovalisporum Biovolumes

To measure the strength of the linear relationship between *Chrysosporum ovalisporum* biovolumes and cylindrospermopsin concentrations, Pearson’s correlation coefficients were computed on 31 samples taken at 0, 5 and 10 m depths in 2012 and 2013. A negative low value of *r*^2^ = −0.05 showed the absence of any correlation between CYN concentrations and *Chrysosporum ovalisporum* biovolumes. Although *Chrysosporum ovalisporum* biovolumes in 2013 was eight times lower than in 2012, cylindrospermopsin was 1.72 µg L^−1^, as high as in 2012.

## 3. Discussion

### 3.1. Chrysosporum ovalisporum Blooms in Karaoun Reservoir

Cyanobacterial blooms are a new phenomenon in Karaoun Reservoir compared to the near Lake Kinneret (Israel), located 80 km to the south of Karaoun Reservoir. In Lake Kinneret, winter and spring blooms of *Microcystis* sp. were reported since the 1960s [31]. Also, an intense bloom of *Chrysosporum ovalisporum* occurred for the first time in the summer of 1994 [10]. Although there is no direct evidence for this, viable cells of *Chrysosporum ovalisporum* could have been transported from Lake Kinneret to Karaoun Reservoir by migratory birds and they could have bloomed when environmental conditions became favorable.

Horizontal displacement, nitrogen availability and water temperature could be the factors controlling the growth and succession of *Chrysosporum ovalisporum* in Karaoun Reservoir. In mid May 2012, *Chrysosporum ovalisporum* dominated in samples taken at the edge of the reservoir. Its concentration greatly decreased within 10 days. Profiles of *Chrysosporum ovalisporum* on May 24, 2012 showed that it was concentrated in the top 5 m. Between May 14 and 24, 2012, the reservoir was full and overflowed. Horizontal transport of buoyant *Chrysosporum* by wind and current may have caused their loss after they exit the reservoir through the spillway. Horizontal displacement is considered as the limiting factor that causes the loss of floating colonial cyanobacteria through outflow. It was reported to be the main reason affecting the horizontal distribution of phytoplankton, especially buoyant cyanobacteria, in Lake Taihu [32] and in four Andalusian reservoirs in Spain [33].

Nitrogen limitation is a factor that can promote *Chrysosporum ovalisporum* growth [34]. The bloom of *Chrysosporum* ovalisporum in October 2012 was preceded by a period of very low nitrogen levels and N:P ratios that did not exceed 22:1 during the study period. According to Smith *et al.* (1995), lake water TN:TP ratios below 22:1 favour the dominance of N_2_-fixing cyanobacteria [35]. Similar effects of low N:P ratios have been seen in Lake Kinneret where the invasion of the nitrogen-fixing cyanobacterium *Chrysosporum ovalisporum* was consistent with the trend towards increasing *N*-deficiency in the water column [36]. 

*Chrysosporum ovalisporum* occurred at different water temperatures in other freshwater bodies. In July 1999, *Chrysosporum* ovalisporum dominated at subsurface water temperatures between 29 and 31 °C, in the warm monomictic Lakes Lisimachia and Trichonis in Greece [7]. Laboratory experiments showed that *Chrysosporum ovalisporum* of Lake Kinneret has an optimal temperature of 26–30 °C [14]. In Arcos Reservoir, *Chrysosporum ovalisporum* dominated in October and September at a subsurface temperature of 26 °C [9]. Unlike the temperature conditions that were associated with blooms of *Chrysosporum ovalisporum* in Lakes Kinneret, Lisimachia, Trichonis and Arcos Reservoir, *Chrysosporum ovalisporum* in Karaoun Reservoir peaked in October 2012, with a maximum biovolume of 9.8 mm^3^ L^−1^, when water temperature was 22 °C. Although there is a difference in the water temperature at which *Chrysosporum ovalisporum* blooms in Lake Kinneret and Karaoun Reservoir, climate change is thought to be one of the drivers of *Chrysosporum ovalisporum* blooms. Slim *et al.* [29] revealed that changes in climate regime, increase in air temperature and decrease in precipitation between 2000 and 2010 have altered Karaoun Reservoir biodiversity and resulted in low diversity dominated by *Chrysosporum ovalisporum* and *Microcystis aeruginosa* blooms. In Lake Kinneret, Hadas *et al.* [37] proposed that the appearance and establishment of *Chrysosporum ovalisporum* since 1994 was linked to increased water temperature and limited nitrogen availability. Using a temperature based model, Mehnert *et al.* [16] hypothesized a future northward expansion of *Chrysosporum ovalisporum* in Europe under the global warming scenario. In Karaoun Reservoir, *Chrysosporum ovalisporum* bloomed at a water temperature of 22 °C. This supports the possibility of *Chrysosporum ovalisporum* blooms in European lakes in which subsurface water temperature can exceed 22 °C like Lake Bourget in France [38], Lake Mondsee in Austria [39], and Lake Zurich in Switzerland [40].

As in Cobaki Village Lake in Australia [41], *Chrysosporum ovalisporum* in Karaoun Reservoir was present in the epilimnion with the highest cell concentrations occurring at a depth of 1 to 3 m in spring 2012 when irradiance was 250 ± 20 W m^−2^. Its highest filament concentrations then occurred at top 1 m when irradiance was 100 ± 20 W m^−2^. *Chrysosporum ovalisporum* in Karaoun Reservoir is probably also sensitive to photoinhibition as in Lake Kinneret where the rate of photosynthesis of *Chrysosporum ovalisporum* reaches maximum at about 80 W m^−2^ and declines at higher irradiance, due to photoinhibition [14].

### 3.2. Relation between Cylindrospermopsin Concentrations and Chrysosporum ovalisporum

CYN can be present in water body as extracellular and intracellular. The extracellular fraction can exceed the intracellular fraction [20]. The low correlation between CYN concentrations and *Chrysosporum ovalisporum* biovolumes can be explained by the ability of this cyanobacterium to liberate high levels of CYN that remains stable even after the decline of the cyanobacterium. CYN is relatively stable under a variety of conditions; it decomposes slowly in temperatures ranging from 4 to 50 °C at pH 7. After 10 weeks at 50 °C, cylindrospermopsin degraded to 57% of the original concentration [24]. According to Preußel *et al.* [23] several temperature–light combinations which constitute physiological stresses seem to trigger CYN production and particularly CYN release from cells. Shaw *et al.* [42] found that the extracellular cylindrospermopsin fraction was at least 85% in Ocean Park ponds and Palm Lakes in Australia, indicating that *Chrysosporum ovalisporum* releases cylindrospermopsin to water. For that, analyses based on *Chrysosporum ovalisporum* cell counts cannot decipher cylindrospermopsin concentration because they do not take into account extracellular CYN.

### 3.3. Disappearance of CYN from Water Column by Degradation or Sedimentation

Vertical profiles of CYN in Karaoun Reservoir showed that its concentration decreased at the surface and increased at deeper depths during summer. Information about the vertical distribution of CYN and its disappearance from the water column in other freshwater bodies are scarce. Settling after adsorption to particulate material or degradation may have resulted in the disappearance of CYN from the surface.

*In situ* photodegradation of CYN was observed, but rate is affected by both the turbidity of the water and the depth of the photic zone [24]. Little information is available regarding the effect of temperature on the biodegradation of cyanobacterial toxins [43]. There are conflicting reports regarding the efficiency of the biodegradation of these metabolites in water bodies [43]. For example, Smith *et al.* [44] documented biodegradation in water supplies that had a history of toxic *Cylindrospermopsis raciborskii* blooms in North Pine Dam in Queensland, Australia, while Wormer *et al.* [45] did not observe any biodegradation of cylindrospermopsin produced by *Chrysosporum ovalisporum* in Santillana Reservoir in Spain. The profiles presented on Figure 6 represent both intracellular and extracellular CYN. A large fraction of CYN was in extracellular form when it started to decrease at 1 m depth because *Chrysosporum ovalisporum* was not detected. Extracellular toxins may adsorb to clays and organic material in the water column [46]. The settling velocity of CYN was about 1 m per week which means that it might have been adsorbed on organic material rather than clay that needs months to settle. In Cobaki Village Lake, Australia, the maximum toxin concentration was present in the hypolimnion during a *Chrysosporum ovalisporum* bloom [41]. This suggests that the decrease of CYN concentration at surface in Karaoun Reservoir might de due to CYN settling or degradation.

## 4. Experimental Section

### 4.1. Study Site

**Figure 7:**
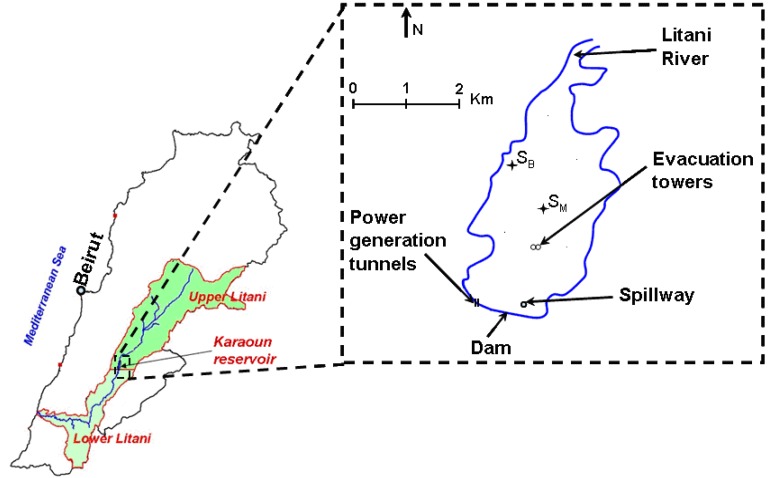
Karaoun Reservoir and sampling sites: S_M_ (33°34'05''N, 35°41'44''E) and S_B_ (33°34'37''N, 35°41'20''E).

Karaoun Reservoir (33°34'N, 35°41'E), located in the southern part of the Bekaa valley, between the two Lebanese mountain chains, is the largest freshwater body in Lebanon (Figure 7, Table 2). The reservoir was constructed between 1958 and 1965 on the Litani River for power production and irrigation. Most of the river inflow (90% of the mean annual inflow) occurs mainly in the wet season, from October to May, while the withdrawals are regular throughout the year, which causes a large level variation, by about 20 m [47]. 

**Table 2:** Karaoun Reservoir morphometric and hydrologic characteristics.

Morphometry and hydrology	Values
Surface area at full capacity	12 km^2^
Maximum storage capacity	224 × 10^6^ m^3^
Maximum depth	60 m
Mean depth at full capacity	19 m
Altitude at maximum level (m)	860 m
Catchment area	1600 km^2^
Mean residence time of water	9 months

### 4.2. Sampling Procedure

Measurements and samples were taken at the most representative point (S_M_), located in the middle of the lake (33°34'05''N, 35°41'44''E). However, for safety reasons during high water level, the sample of May 15, 2012 was taken at the reservoir side, S_B_ (Figure 7). Campaigns were performed bi-weekly between 11:00 and 13:00. Water samples were collected at 0.5 m depth from May to November 2012 and at 0.5, 5 and 10 m depths from March to August 2013 with a vertical Niskin bottle of 2.2 L capacity (Wildco 1120-D42, Florida, United States). Samples were stored at 4 °C until further processing in the laboratory. Different volumes and bottles were used for phytoplankton identification and counting, nutrient analysis and cylindrospermopsin quantification.

### 4.3. Water Temperature and Phycocyanin Measurements

Water temperature was measured with temperature sensors (Starmon mini, Star-Oddi, Gardabaer, Iceland) at 1 and 10 m depths to monitor thermal stratification. The sensor measuring temperature range is −2 to 40 °C with an accuracy of ±0.05 °C. A submersible fluorometer (TriOS microFlu-blue, Rastede, Germany) was used to measure fluorescence profiles of phycocyanin, a pigment specific to cyanobacteria. It is equipped with ultra-bright red LEDs, of an excitation wavelength 620 nm, detection wavelength 655 nm and band-width 10 nm. It gives a linear response to phycocyanin concentration up to 200 µg L^−1^ with an accuracy of 0.02 µg L^−1^. Measurements were performed every half meter between the surface and the bottom of the reservoir by descending the cable manually at a speed of 5 cm s^−1^ and waiting for 30 s for values to become stable.

### 4.4. Microscopic Identification and Counting

The subsamples used for phytoplankton counting were fixed by formaldehyde and preserved at 4 °C (4% of sample volume). The phytoplankton species were determined on the sampling day according to taxonomic keys based on cell structure and dimensions, colony morphology, and mucilage characteristics [48,49]. Microscopic identification and enumeration were carried out under a phase contrast microscope (Nikon TE200, Nikon, Melville, NY, USA), under a ×40 objective and using Nageotte chamber that accepts 100 µL on 40 bands. The number of bands counted depended on sample concentration. Each subsample was counted on triplicate.

### 4.5. Nutrient Analysis

Subsamples used for the analysis of nutrients (total phosphorus, orthophosphate, nitrate, and ammonium) were preserved at 4 °C after addition of 2 mL of 18 M H_2_SO_4_ Soluble phosphorus (orthophosphate), nitrate, and ammonium subsamples were then filtrated through a 0.45 µm cellulose acetate filter (MF-Millipore, HAWP04700, American Fork, UT, USA).

Nitrate and ammonium concentrations were estimated by colorimetry with a photometer (Palintest Photometer 7000se, Gateshead, UK). Total phosphorus and orthophosphate concentrations were determined at 880 nm by UV/VIS spectrophotometer (Thermospectronic, LaboTech, Beirut, Lebanon) using colorimetric ascorbic acid method (EPA Standard Method 365.3, Washington, DC, USA). The quantification range for nitrate nitrogen is 0.1–30 mg N L^−1^, ammonium nitrogen 0.1–12 mg N L^−1^, and phosphorus 0.01–1.2 mg P L^−1^.

### 4.6. Cylindrospermopsin Analysis

Samples for cylindrospermopsin analysis were collected in borosilicate bottles, transported in the dark and preserved at 4 °C until analysis date. To measure the concentration of both intracellular and extracellular toxin forms, samples were vortexed before analysis but not filtered. According to Humpage *et al.* [50], high amounts of cyanobacterial cell material or a relatively high organic load, even in wastewater, do not have a significant effect on the analysis result. ELISA kit (Abraxis, product number: 522011, Warminster, UK) was used to evaluate extracellular cylindrospermopsin concentration. Each sample was run in triplicate. The absorbance of the coloured product of antibody-conjugated enzymes was read at 450 nm using a microplate ELISA photometer (Stat Fax 303 Plus, Palm City, FL, USA). The subsequent quantification was based on calibration curves of the semi-logarithmic relationship between relative absorbance and toxin concentration using the six standards provided with the kit. The quantification range for cylindrospermopsin by ELISA is 0.04–2 µg L^−1^.

### 4.7. Meteorological and Hydrological Data

Solar radiation, precipitation and wind speed measurements were obtained from Tal-Amara meteorological station of the Lebanese Agriculture Research Institute located in the Bekaa valley (33°51'50''N, 35°59'06''E), 40 km North of Karaoun Reservoir. Daily water level measurements were provided by the Litani River Authority (LRA), responsible for the management of the reservoir.

## 5. Conclusions

*Chrysosporum ovalisporum* in Karaoun Reservoir was detected in all seasons. It is difficult to decide conclusively its relationship with all the environmental factors and nutrient availability in particular. *Chrysosporum ovalisporum* dominated both during periods of high and low water level, stratification and mixing in a wide range of light irradiances and water temperatures. Light irradiation higher than 250 W m^−2^ photoinhibited *Chrysosporum ovalisporum* and resulted in its concentration between 1 at 3 m depths. Among the main hypotheses for explaining its decline in Karaoun Reservoir is water temperature higher than 25 °C and horizontal transport and withdrawal.

Unlike high temperature conditions which were associated with blooms of *Chrysosporum ovalisporum* in lakes located in the Middle-East or in Southern Europe, *Chrysosporum ovalisporum* in Karaoun Reservoir bloomed at water temperature of 22 °C. Lakes in which water temperature exceeds 22 °C are susceptible to blooms of *Chrysosporum ovalisporum*. Our results suggest that within a period of a month, CYN produced by *Chrysosporum ovalisporum* disappeared from the water column either by sedimentation or degradation. It also shows that CYN concentrations were not correlated with *Chrysosporum ovalisporum* biovolumes and it can be observed at high concentrations even long after the end of *Chrysosporum* ovalisporum blooms.

On the local level, it shows that Karaoun Reservoir contains cylindrospermopsin. Presence of cylindrospermopsin long after blooms of *Chrysosporum ovalisporum* requires regular monitoring of cylindrospermopsin in the Reservoir, to avoid health problems when using Karaoun water for irrigation or for drinking water supply.
